# Genomic lineages of *Rhizobium etli *revealed by the extent of nucleotide polymorphisms and low recombination

**DOI:** 10.1186/1471-2148-11-305

**Published:** 2011-10-17

**Authors:** José L Acosta, Luis E Eguiarte, Rosa I Santamaría, Patricia Bustos, Pablo Vinuesa, Esperanza Martínez-Romero, Guillermo Dávila, Víctor González

**Affiliations:** 1 Centro de Ciencias Genómicas, Universidad Nacional Autónoma de México, Av. Universidad N/C Col. Chamilpa, Apdo. Postal 565-A, Cuernavaca 62210, México; 2 Departamento de Ecología Evolutiva, Instituto de Ecología, Universidad Nacional Autónoma de México, CU, AP 70-275 Coyoacán, 04510 México, DF, México

## Abstract

**Background:**

Most of the DNA variations found in bacterial species are in the form of single nucleotide polymorphisms (SNPs), but there is some debate regarding how much of this variation comes from mutation versus recombination. The nitrogen-fixing symbiotic bacteria *Rhizobium etli *is highly variable in both genomic structure and gene content. However, no previous report has provided a detailed genomic analysis of this variation at nucleotide level or the role of recombination in generating diversity in this bacterium. Here, we compared draft genomic sequences versus complete genomic sequences to obtain reliable measures of genetic diversity and then estimated the role of recombination in the generation of genomic diversity among *Rhizobium etli*.

**Results:**

We identified high levels of DNA polymorphism in *R. etli*, and found that there was an average divergence of 4% to 6% among the tested strain pairs. DNA recombination events were estimated to affect 3% to 10% of the genomic sample analyzed. In most instances, the nucleotide diversity (π) was greater in DNA segments with recombinant events than in non-recombinant segments. However, this degree of recombination was not sufficiently large to disrupt the congruence of the phylogenetic trees, and further evaluation of recombination in strains quartets indicated that the recombination levels in this species are proportionally low.

**Conclusion:**

Our data suggest that *R. etli *is a species composed of separated lineages with low homologous recombination among the strains. Horizontal gene transfer, particularly via the symbiotic plasmid characteristic of this species, seems to play an important role in diversity but the lineages maintain their evolutionary cohesiveness.

## Background

Bacterial species typically contain large amounts of genetic variation in the form of single nucleotide polymorphisms (SNPs), which originate by mutation and have dynamics that depend on the balance between natural selection and genetic drift [1,2]. There is some debate on whether or not most of these polymorphisms are selectively neutral at the molecular level [3]. Species have been genetically defined through the analysis of DNA variation using comparative techniques such as hybridization, the sequencing of gene markers, and (more recently) complete genome sequences [4,5]. It has been proposed that similarity values greater than 70% obtained in DNA-DNA hybridization experiments are sufficient to define a coherent group of organisms as belonging to the same species [6]. These estimates are very rough, subject to experimental variation, and they only indirectly measure similarity (i.e. via hybridization efficiency) [7]. A comparative analysis of complete genomes minimizes most of these limitations. Several measures of genomic relatedness, such as the Average Nucleotide Identity (ANI) and the Maximal Unique Matches (MUM) have been proposed for such analyses [8,9]. Both ANI and MUM are based on pairwise nucleotide comparisons of complete genomes, and several reports have shown good correlations between the results from these analyses and other measures of genetic relatedness, such as those based on Multilocus Sequencing Typing (MLST), 16S sequencing, and gene content [10]. However, these comparative methods rely on the availability of complete genome sequences and are affected by the quality of the DNA sequencing data, which in the case of draft genomes might not be optimal [10]. The latter issue has not been thoroughly addressed in past studies. One exception was the comparisons made by Richter and Roselló-Mora [10], who suggested that low genome sequence coverage can be sufficient for inferring DNA similarity values comparable to ANI obtained with complete genomes.

Bacterial species have mechanisms for gene exchange (transformation, conjugation and transduction), and genetic recombination is believed to play a prominent role in diversifying species by distributing variation and generating new allele combinations [11]. Horizontal gene transfer is an important source of genomic variation within and between species [12-16], and homologous recombination frequently results in the exchange of small genomic regions between members of the same or closely related species [17]. The estimated rates of homologous recombination vary widely among bacteria; in some instances, recombination seems to have contributed to species diversification to a greater extent than even point mutations, whereas in other species homologous recombination appears to be rare [18].

Recombination has typically been assessed by molecular techniques such as Multilocus Enzyme Electrophoresis (MLEE), Amplified Fragment Length Polymorphism (AFLP), or Multi Locus Sequence Typing MLST [19-21]. These methods primarily measure linkage disequilibrium (LD), and are based on the degree of allele association at different housekeeping loci. For example, *E. coli *strains show strong LD, reflecting infrequent genetic mixing within local populations [22]. More recently, the availability of complete genomic sequences has allowed recombination to be assessed more accurately [23]. Interestingly, genomic sequencing combined with analyses of population genetics have shown that the recombination rates within *E. coli *are higher than the mutation rates, but not to the extent that the phylogenetic signal is distorted [24]. Despite frequent recombination between strains, therefore, the genes seem to coexist in an organized genome, resulting in a chromosomal plasticity that accelerates the adaptation of *E. coli *to various environments.

In this work, we studied the intraspecific variability and recombination in *Rhizobium etli*, a soil bacterium that associates with bean roots to fix nitrogen. Previous studies have noted that this species has a variable gene content and high genomic divergence [24], as well as a low rate of recombination (in housekeeping genes) among isolates from the same geographical site [22,25,26]. However, in isolates (from the same geographical site) of *Sinorhizobium medicae*, it was found that frequency of recombination was higher in plasmids and megaplasmids, as compared to the chromosome [27]. The first purpose of this work was to perform a detailed genomic analysis of the nucleotide variation in this species. Accordingly, we used stringent methods to identify SNPs from a set of complete and draft genomes of *R. etli*, assessed the value of draft genomes and low coverage data when seeking to obtain global measures of genetic relatedness, and then examined the nucleotide differences among various strains of *R. etli*. The second purpose was to assess the role of recombination in generating genomic diversity in *R. etli*. Our results confirm and extend the previous estimations on the genomic diversity of *R. etli*, and indicate that recombination might play only a minor role in generating such diversity. Therefore, we conclude that the species *R. etli *is composed of separate genomic lineages that share a low rate of recombination but have a common symbiotic phenotype.

## Results

### Nucleotide variation assessment in complete and draft genomes

Since accurate SNP identification relies largely on the quality of the sequence data, the use of draft genome sequences could potentially introduce errors into the variation estimates. Therefore, stringent parameters (see Methods) were used to identify high-quality SNPs in a set of two complete *R. etli *genomes, CFN42 and CIAT652, isolated from México and Costa Rica respectively, and six draft genome sequences from strains isolated in different places of the world: BRASIL5 (Brazil), CIAT894 (Colombia), GR56, IE4771 (México), KIM5 (USA), and 8C-3 and GR56 (Spain) [24]. All the Sanger reads were collected from the draft genomes (about 13,000 reads of 1000 nucleotides in length per genome on average) were aligned against the predicted ORFs of the CFN42 or CIAT652 genomes, and the alignments were evaluated using Polybayes (additional file 1 Figure S1), which determined the probability that a nucleotide site was polymorphic, based on the Phred quality of the read. A Phred value of Q20 and a probability greater than 0.90 are generally considered acceptable for the detection of SNPs [28]. Most of the SNPs in our data set had probability scores > 0.975, indicating that more than 100,000 SNPs per genome had Phred qualities over Q45 (additional file 1 Figure S1). To avoid the possible inclusion of false positives (in average 27,000 SNPs by each strain), we used only SNPs with a minimum Phred score of Q45 and the highest Bayesian probabilities (> 0.99) throughout this work [29].

Additional errors in SNP determination might arise from poorly aligned regions. Since *R. etli *genomes have a high proportion of paralogous sequences [24,30], a stringent identification of orthologous segments of genes was performed. We aligned the contigs of each draft genome sequence against the ORFs from the complete genomes of either CFN42 or CIAT652, using both ungapped and gapped alignments, along with the reciprocal best hit criteria. We considered DNA gene segments as being orthologous to the reference sequence if they had nucleotide identities higher than 85% and coverage higher than 60% of the reference gene. Various numbers of orthologous segments were identified from the draft genomes, covering about 40% of the total gene contents of the reference strains. The total amount of data collected by this procedure is about 2 to 2.5 Mb per draft genome (additional file 1 Table S1).

**Figure 1:**
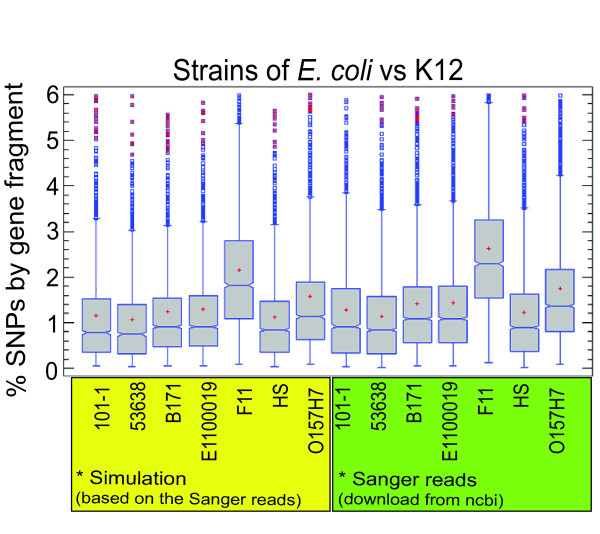
**SNP assessment on lower coverage**. Paired comparisons between *E. coli *strains and *K12 *strain as reference genome. We used two set of *E. coli *strains: Complete genomes obtained from Sanger reads (green box) and Sanger reads simulated from complete genomes with coverage approximate of 1X (yellow box). For each alignment, we determined the percentage of SNPs by gene fragment (SNP number/length of contig; Y axis) by our methodology. Boxes inside the graphic include the median values (middle line) and the first and third quartiles (lower and upper lines) of the distribution. Abscissa: *E. coli *strains.

To determine the robustness of the above-described procedure, we simulated a draft assembly by using Sanger read samples of the complete genomes of different *E. coli *strains at low coverage (1x) (see Methods). The contigs of the simulated assembly were aligned with the genome of *E. coli *K12, and SNPs were detected as described above. On average, the obtained nucleotide variation ranged from about 1% to 2% (SNPs/alignment length) (Figure 1). There was no significant difference (p-value lower at 0.05, according to Mann-Whitney and Kolmogorov-Smirnov tests obtained from Predictive Analytics Software PASW Statistics 18 (SPSS Inc., Chicago, IL)) when we compared the results obtained at 1× coverage versus those obtained with the complete genome assembled at about 10× coverage, indicating that 1× coverage of the genome sequence could be considered a robust proxy of full variation at the genomic level in this species.

### SNP frequencies among the *R. etli *strains

**Figure 2:**
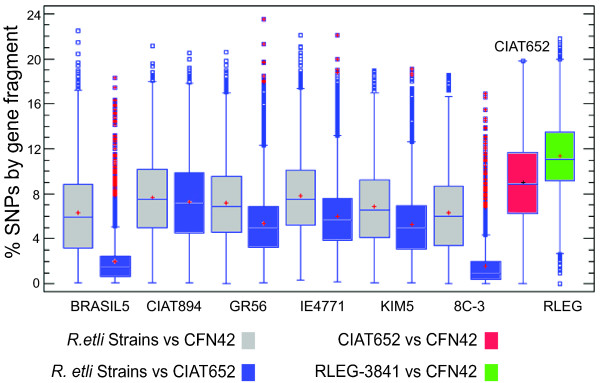
**Paired comparisons between *R. etli *strains**. We performed four paired comparisons used our methodology: draft genomes of *R. etli *against CFN42 (gray boxes), draft genomes of *R. etli *against CIAT652 (blue boxes), CIAT652 against CFN42 (red boxes) and finally *R. leguminosarum bv viciae 3841 *against CFN42 (green boxes). For all comparisons the Y axis is the percentage of SNPs by gene fragment (SNP number/length of contig). Boxes inside the graphic include the median values (middle line) and the first and third quartiles (lower and upper lines) of the distribution. Abscissa: *R. etli *and *R. leguminosarum bv viciae 3841 *strains.

We quantified the SNPs in *R. etli *by computing the pairwise nucleotide differences between individual draft genomes versus the complete genomes of strains CFN42 or CIAT652. More SNPs were found in comparisons made versus the CFN42 genome (Figure 2, gray boxes) than the CIAT652 genome (Figure 2, blue boxes). For example, the BRASIL5 strain had a median of 5% SNPs per aligned fragment when compared with CFN42 but only 2% compared to CIAT652, indicating that BRASIL5 is more closely related to CIAT652 than CFN42. Similarly, variance was higher when BRASIL5 was compared with CFN42 rather than CIAT652 (Figure 2). A very similar pattern was found for strain 8C-3. The other strains showed similar levels of variation, on the order of 6% (CFN42) and 4% (CIAT652), with the latter comparison always showing a lower variance. Comparison between the complete genomes of CFN42 and CIAT652 (Figure 2, red box) result in a median variation of 9%, that is high but still lower than the comparisons between CFN42 and *R. leguminosarum *bv *viciae *3841 (Figure 2 green box). Moreover, when we compared *R. leguminosarum bv. viciae *3841 with all of the *R. etli *strains (complete and draft genomes) (additional file 1 Figure S2), the greatest difference in SNP percentage (median 11%) was seen in the comparison with strain CFN42 (Figure 2 green boxes, and discussion section).

### Average nucleotide variation

**Figure 3:**
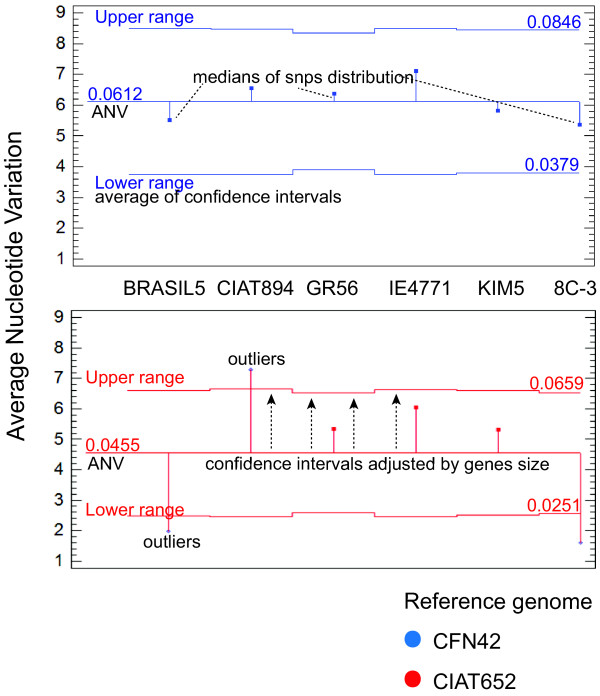
**Average nucleotide variation**. We calculated the Average nucleotide variation (middle lines of each graphic) from the median SNP percentages (dots indicates by dashes lines) for each aligned comparison (Y axis) of test strain versus the reference strains, CFN42 (blue) or CIAT652 (red). Average Confidence interval was adjusted (arrows with dashes lines) to the medians of the length distributions of the aligned fragments (genes). The medians SNPs that exceed the average confidence interval were outliers. Abscissa: BRASIL5, CIAT894, GR56, IE4771, KIM5 and 8C-3.

We sought to obtain a single measure of the nucleotide variation across the whole set of genomes. To this end, we averaged the medians of the SNP distributions for each alignment (*i.e.*, the number of SNPs/alignment length of each draft genome with respect to CFN42 or CIAT652) and generated average confidence interval (obtained and adjusted by distribution of genes size medians) using Predictive Analytics Software PASW Statistics 18 (SPSS Inc., Chicago, IL). This statistical test of proportions compares the observed proportions of an event (here, SNPs) in k samples (here, strains), uses a chi-squared test to seek significant differences among the proportions, and subsequently adjusts the confidence intervals for each sample. The generated measure, herein called the average nucleotide variation (ANV), might represent the species-level variation. We obtained ANV values of 4% and 6% when we compared all the analyzed strains against CIAT652 and CFN42, respectively (Figure 3). Although the largest numbers of SNPs were found in comparisons with the CFN42 genome, all strains were similarly divergent according to the 95% confidence intervals with respect to the median (blue lines in Figure 3). This observation indicates that CFN42 is almost equally divergent with respect to all other strains. Comparisons with the CIAT652 genome showed that strains BRASIL5 and 8C-3 were closer to this strain than to CFN42. Moreover, the CIAT894 strain yielded the highest number of SNPs, causing its average SNP proportion to fall outside the average confidence interval (red lines in Figure 3). Strains CIAT894 and IE4771 showed greater divergences than the rest of the strains, regardless of the reference strain (CFN42 or CIAT652) used in the comparison.

### Nucleotide variation profiles in homologous genomic segments from different *R. etli *strains

**Figure 4:**
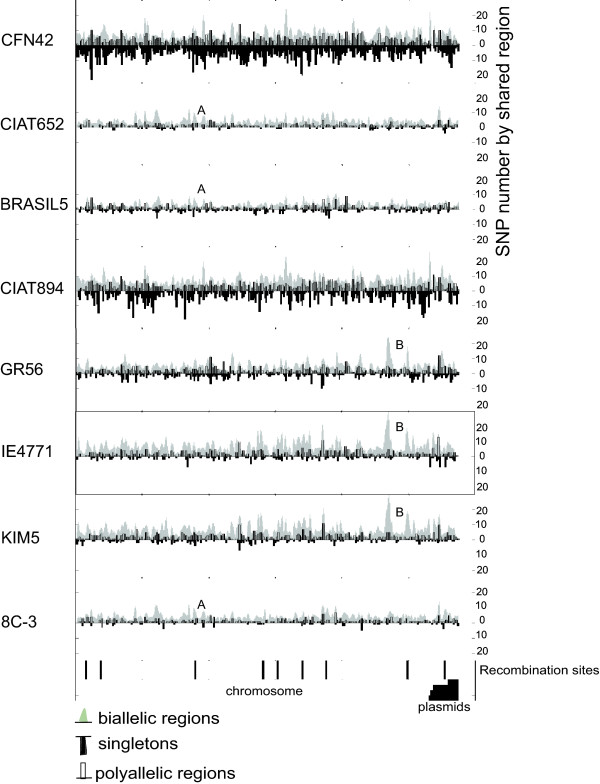
**SNP distribution profiles**. Alignments were performed on a total of 240 sequence segments available for all tested strains of *Rhizobium etli*. Each nucleotide position in the alignment is represented by a consensus. In instances where half of the strains had the same nucleotide and the other half a different nucleotide, the consensus was defined as the nucleotide present in *R. etli CIAT652*. Common segments were concatenated according to the gene order found in the CFN42 genome (chromosome and after plasmids), yielding 71,630 aligned base pairs. The numbers of nucleotides differing from the consensus are plotted as bars, across independent windows of 250 nucleotides. The black bars (running downwards) show SNPs present in a single strain; the gray areas indicate when the same SNP pattern was present in at least two strains at the same position within the alignment (patterns A and B); and the white bars indicate polymorphic sites where at least three alleles were present in at least two strains, again within the alignment. Segments showing significant recombination events are indicated by bars at the bottom of the plot, and with bars indicating the genomic location of segments with respect to CFN42 (chromosome, white; plasmids, black).

To explore how SNPs are distributed in the *R. etli *genomes, we first identified orthologous segments for which we had sequence information in all eight studied strains (Figure 4). A total of 240 segments with a median size of 275 bp were common to all strains, and spanned a total of about 71,630 bp that represent about 1% of the genome length. These sequences mapped mainly to the chromosomes of CFN42 and CIAT652 (92%), with a lower proportion (8%) distributing to plasmids. We generated a concatenated alignment of these shared segments according to the gene order found in the CFN42 genome, and then inferred a consensus sequence and computed the number of nucleotide differences across windows of 250 bp. Using this procedure, we detected the patterns of shared and unique (singleton) SNPs particular to each strain. As shown in Figure 4, we were able to distinguish two classes of shared SNPs: biallelic SNPs (Figure 4 gray smoothed areas), which showed only one nucleotide difference with respect to the consensus; and polyallelic (Figure 4, white bars), which showed multiple differences at the same nucleotide site with respect to the consensus. Some of these SNP patterns were shared in some strains but not others. For example, as shown in Figure 4, pattern A was shared by strains CIAT652, CIAT894 and 8C-3, whereas pattern B was found in strains GR56, IE4771 and Kim5. Further shared patterns were identified through a careful inspection of the plot. In addition, a large number of polymorphisms were not shared, but instead appeared to be strain-specific variants. Interestingly, strain CFN42 was found to have the greatest number of differences with respect to the consensus (Figure 4, black bars). Even thought this approach is limited by the amount of common segments among the eight strains, we were able to cover 3.7% (223) of the total gene content (5,963) of the CFN42 reference strain that include the main COG categories and subcategories (see Methods). For instance, metabolism (transport and metabolism of sugar, amino acids, and carbohydrates); cellular processes and signaling (envelope biogenesis, signal transduction); information storage and processing (transcription, replication, and recombination); and poorly characterized proteins (function unknown). A detailed annotation of the gene segments can be seen in additional file 2 Table S1.

### Phylogenetic congruence

**Figure 5:**
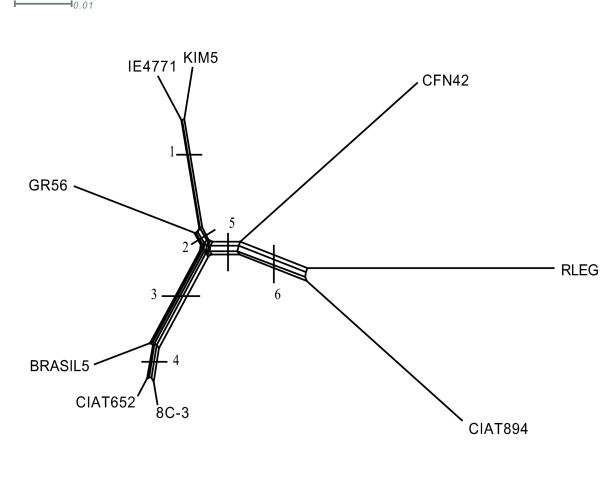
**Genetic relatedness**. Network joining network phylogeny inferred from 186 concatenated regions shared among strains of *Rhizobium etli *and *Rhizobium leguminosarum bv viciae *3841 (see Methods). The tree is unrooted and has six internal branches, indicated by split numbers on each internal branch. The scale bar denotes the expected number of nucleotide substitutions per site.

Since recombination can distort phylogenetic trees such a way that no two individual trees are topologically equivalent, we decided to perform phylogenetic reconstructions using a) a neighbor-joining network [31]; and b) a comparison of a consensus tree with individual trees constructed using the 187 segments common to the eight studied *R. etli *genomes and *R. leguminosarum bv viciae 3841 *(RLEG). The consensus trees obtained from the concatenated alignments had identical topologies when constructed by maximum likelihood, Bayesian, and neighbor joining network methods (see Methods). Only the tree based on neighbor joining network is shown in Figure 5. This tree was found to contain six internal branches (denoted by split numbers). There are two main clusters in the tree, separated by branches 2 and 3 that group the most closely related strains: one containing KIM5, IE4771, and GR56 (branch 2) and another grouping BRASIL5, 8C3, and CIAT652 (branch 3). These branches are internal in relation to branch 5, which separates CFN42, CIAT894, and RLEG that are the strains with the longest branches (greatest number of nucleotide substitution per site). A few inconsistencies were found among the topologies recovered from reconstructions based on individual gene segments (187), as compared to the topology of the consensus tree (not shown). These alternative topologies are mainly due to the position of CIAT894 and RLEG, whereas the splits 2, 3, and 5 where consistently recovered. Thirty out of 187 trees supported the placement of RLEG as the most distant strain, 39 trees supported placement of CIAT894 as the external strain, whereas the most frequent topology shows that these strains are equally distant to the rest of strains (Figure 5). These alternative topologies could be the result of shared ancestral polymorphisms, as suggested by the long branches coupled with low frequency of recombination. Altogether, the phylogenetic reconstructions suggested that the levels of recombination were insufficient to erase the phylogenetic signal, thus allowing for the identification of the most probable strain tree. Consistent with this conclusion, only nine (3.75%) of the 223 gene segments common among the eight *R. etli *strains (Figure 4) showed at least one recombination event.

### Extent of recombination

**Figure 6:**
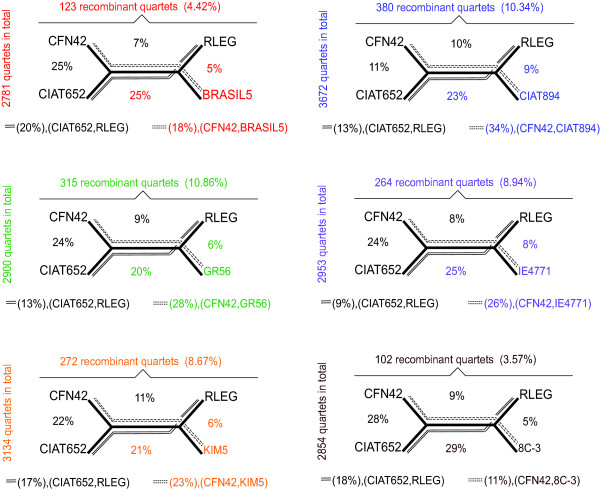
**Detection of recombination in quartets**. Six groups of quartets of orthologous segments were built with the Mauve program; they included shared sequences from CFN42, CIAT652, *Rhizobium leguminosarum bv viciae 3841*, and each one of the six test strains (incomplete genomes). Test of recombination were performed for all the quartets as described in Methods section. The number (as well as the percentage) of recombinant segments predicted for each group of quartets is indicated above the quartet diagram. The total number of quartets analyzed in each group is indicated at the left side of the diagrams. The percentages of recombinant quartets between pairs of strains are shown inside the diagram and by dashed and continuous lines defined below the diagram. To facilitate searching each test strains o incomplete genome has its own color.

To evaluate the extent of the probable recombination events among strains of *R. etli*, we performed a recombination analysis in orthologous quartets (see Methods). We aligned the shared gene segments from each draft genome with the corresponding segments of the ORFs from CFN42, CIAT652, and the *R. leguminosarum bv viciae 3841 *complete genomes, yielding six different groups of quartets (one group for each incomplete genome; Figure 6). The proportion of aligned segments varied across the six groups of quartets, from ~2,781 segments in the group containing BRASIL5, to ~3,672 in the group containing CIAT894. The segments ranged from 200 to 4651 bp in length and covering approximately 50% of the genome (additional file 1 Table S2). For each group of quartets, we performed four different recombination tests (see Methods), and determined the number of recombination events (only those that were detected by at least two methods) for each quartet (describe above) (Figure 6). The lowest proportions of recombination events were detected for the quartets containing strains BRASIL5 and 8C-3, which showed 4.42% (123 out 2781) and 3.57% (102 out 2854) recombination events, respectively. The other groups showed approximately twice as many recombination events, with frequencies ranging from 8.67% (KIM5 quartets) to 10.86% (GR56). In addition, for each group of recombinant quartets, we determined the number of events of recombination between pairs of strains (Figure 6). In general, recombination events were more frequently predicted between *R. etli *strains pairs than between any given *R. etli *strain and *R. leguminosarum bv viciae 3841 *(Figure 6). For instance, in the group of quartets containing BRASIL5, the percentage of recombinant segments is about 7% in CFN42-RLEG, 5% in BRASIL5-RLEG, and 20% in CIAT652-RLEG pairs, whereas recombinant segments were detected more frequently between pairs of *R. etli *strains: 18% (CFN42-BRASIL 5), 25% (CFN42-CIAT652), and 25% (CFN42-CIAT652). The same pattern was seen for the other five groups of quartets. This effect is because homologous recombination depends on a high nucleotide identify, and greater divergence is associated with less homologous recombination [32]. Therefore, recombination might be more frequent between strains (populations) that are closely related. Indeed, we observed the same recombination events in different groups of quartets (of different strains), as indicated by a presence/absence matrix. In general, the number of common recombination events (small number of events) was related to the phylogenetic proximity of the strains, for instance BRASIL5 and 8C-3 share the most recombination events in common (data not shown).

To explore whether the recombination is particularly acting on some classes of genes, we assigned the recombinant segments to COGs (see Methods), as shown additional file 1 Figure S3. All the functional classes annotated in the CFN42 genome are present in the draft genomes but they are represented unevenly in the recombinant segments. For instance, the categories: amino acid transport and metabolism, carbohydrate transport and metabolism, energy production and conversion, lipid transport and metabolism, general function prediction only and function unknown appear overrepresented among the recombinant segments. In counterpart, some other categories like transcription and signal transduction mechanisms are in lower frequency among the recombinant segments than in CFN42. Even though we performed a chi-square and Range tests [33] to assess the significance of these differences, the incomplete nature of draft genomes does not allow to conclude about some bias toward recombination in certain classes of genes.

### Genetic diversity

**Figure 7:**
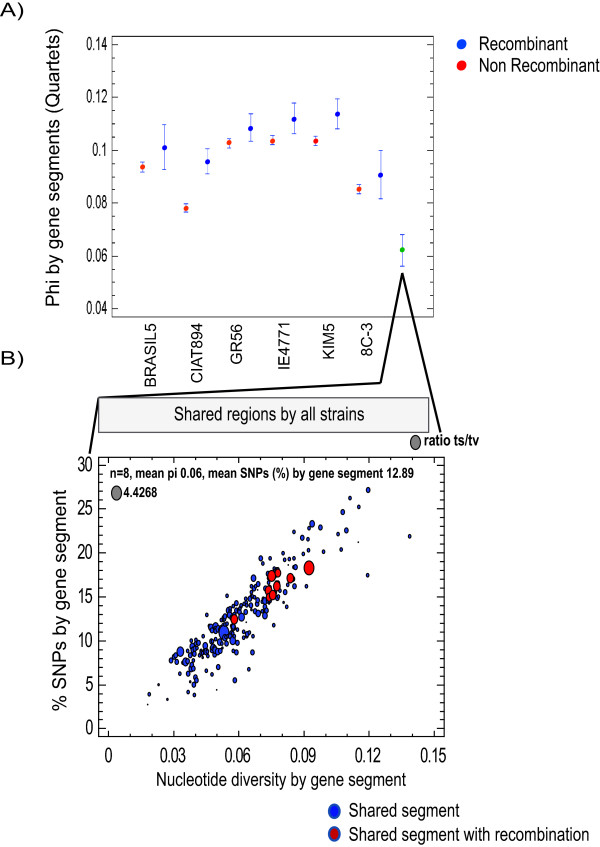
**Genetic diversity in recombinant segments**. A) For each homologous segment (quartet; regardless of evidence of recombination), we calculated the nucleotide diversity phi (Y axis; see Methods). The dots indicate the distribution means and the bars represent the 95% confidence intervals. Blue and red dots indicate recombinant and non-recombinant segments, respectively. Moreover, we determined the nucleotide diversity of the sequence regions shared across all of the tested strains of *R. etli *(green dot). Abscissa: BRASIL5, CIAT894, GR56, IE4771, KIM5, 8C-3 and shared regions. B) Magnification of the results from the 240 common sequence segments shared by all tested strains. The average percentage of SNPs was 12.89% per segment. The sphere sizes indicate the proportions of ts/tv for both recombinant segments (red circles) and non-recombinant segments (blue circles). The Y axis denotes the SNP percentage by gene segment, while the X axis shows the nucleotide diversity.

Together the above-described data suggest that recombination may not be a major driver of genomic diversification in *R. etli*, but rather might have relatively limited effects. To directly examine this point, we estimated the mean nucleotide diversity per nucleotide site (π) for the recombinant and non-recombinant gene segments of each strain (Figure 7a). In general, recombinant segments showed higher π values than non-recombinant segments. These differences were significant only for strains CIAT894, GR56, IE4771 and KIM5 (Student's *t*-test, p < 0.001), but the combined data for the π values of the 240 recombinant and non-recombinant gene segments common to the eight strains showed the lowest π values (0.06 on average). Although there was no significant difference between recombinant (red circles) and non-recombinant segments (blue circles) with regard to the regions common to all eight strains (Figure 7b), most of the recombinant segments had higher-than-average π values and generally showed the highest transition/transversion ratios (indicated by the size of the circles in Figure 7b). Since the probability of transitions is higher than transversions [34], high ratios of transition/transversion suggest that they were under strong purifying selection, because transitions at the third 'wobble' position are more likely to be synonymous than transversions [35].

## Discussion

In the present work, we used a genomic approach to detect and measure variation in the form of SNPs, and to analyze the contribution of recombination to the genomic diversification of *R. etli *strains. Our results demonstrated that draft genomic sequences samples representing ~1× of the genome can be used to measure variation at the whole-genome level in this species. In *R. etli *we found a great amount of variation (more than 161,998 SNPs) when any draft genome was compared to the complete genomes of CFN42 and CIAT652. To assess the reliability of this method for identifying SNPs, we quantified the SNPs in *E. coli *genomes at 1× and in complete genomes assembled at about 10× coverage. We found the same variation level using either draft or complete *E. coli *genomes, indicating that draft genomes produced estimations of DNA variability comparable to those generated using complete genomes even at only 1× coverage. Richter and Roselló-Mora [10] previously reported on the use of partial sequences representing about 20% of the genomes of several bacterial species to infer reliable values of DNA divergence between strains. The authors of the prior paper showed that ANI values obtained with these samples correlated well with the DDH values, indicating that draft genome sequences are an acceptable data source. At present, the rapid improvement of DNA sequencing technology is allowing researchers to use multiplex sequencing to simultaneously process an increasing number of genomic sequences. These experiments will produce additional draft genome sequences of different qualities, and the approach proposed herein should prove useful for their early analysis.

We identified a higher proportion of SNPs in *R. etli *strains than in *E. coli *strains, and the differences between the various *R. etli *strains and *Rhizobium leguminosarum bv viciae 3841 *ranged from 7% to 11% (median; additional file 1 Figure S2), with the latter figure corresponding to the CFN42 comparison. *R. etli *and *R. leguminosarum *are different species according to 16S comparison; however, they share a common genomic core and are distinguished by variable accessory components (e.g., plasmids) [24,36,37]. Therefore, an ANV range of 7-11% might be a good indicator of speciation within *Rhizobium*. Despite of the variability in ANV among the tested strains of *R. etli *(about 4-6%), none had ANV values comparable to those obtained with respect to *R. leguminosarum*. The levels of ANV were higher for comparisons using CFN42 than those done with CIAT652. For taxonomic purposes, CFN42 is the type strain of *R. etli *[38]. In the present analysis, however, we found that CFN42 was the most differentiated of the studied samples, had the highest proportion of unique SNPs, and clustered as a divergent independent branch when the strain phylogeny was explored. We recently re-sequenced strain CFN42 using Solexa-Illumina technology and compared it with the former complete genome sequence. Very few indels and SNPs (less than 20 SNPs) and no rearrangements were found. Therefore, very small variation can be expected from an in vitro lifestyle. In contrast, most of the strains analyzed were more closely related to CIAT652 than to CFN42. A prior study noted that CIAT652 and CFN42 have a low ANI value (90.44%) [10] and suggested that CIAT652 is improperly classified as *R. etli*. We previously showed that CFN42 and CIAT652 share a very conserved symbiotic plasmid, but have high divergence throughout the rest of their genomes [24]. Given that all isolates of *R. etli *have been recovered from nitrogen-fixing bean nodules, this characteristic would be expected to dominate the classification criteria. The genomic divergence described herein is thus consistent with a model in which the species *R. etli *is composed of divergent genomic lineages that share the symbiotic phenotype conferred by the symbiotic plasmid [24], which is called a common symbiovar [39]. Indeed, our analysis suggests that in some instances, the use of type strains could lead to misleading taxonomic classifications, especially when gene transfer mechanisms are active. *R. etli *is known to have mobile elements such as conjugative plasmids, insertion sequences and bacteriophages [40-42]. Therefore, gene flow and recombination among strains of *R. etli *might be important to the production of genomic diversity, as reflected in its pangenomic structure [24]. However, no prior study has assessed the role of homologous recombination in promoting the genomic diversity of *R. etli*. Earlier works using MLEE or MLST concluded that *R. etli *populations are essentially clonal, with low recombination even in sympatric populations [22,25,26,43]. More recently, Flores *et al. *[44] showed that despite the high conservation of the symbiotic plasmid pSym sequences from a collection of different strains of *R. etli*, some regions shared identical SNP distribution profiles. This observation was interpreted as evidence of homologous recombination. Here, we obtained similar findings for a set of common genomic DNA segments, mainly chromosomal in origin, belonging to eight strains of *R. etli*. Quantification of probable recombination events and the extrapolation of our findings to the whole genome suggested that a minimum of 260 recombination events had occurred in the genome of each strain. Strains CFN42 and CIAT894 were the more variable in terms of SNPs, and the latter also showed the most evidence for recombinant events in our quartet analysis (within the orthologous segments). Even though there were some discrepancies within the clades of the various phylogenetic trees we generated, most of the trees were congruent with the consensus tree. Moreover, although the estimated recombination was correlated with genetic diversity (Figure 7a), it was low overall (3-10%). In comparison, the whole-genome recombination estimates reported for *Rickettsia *and *Streptococcus *were on the order of 18-37% and 28%, respectively [45,46]. These data suggest that only a minor fraction of the *R. etli *genome has undergone recombination, which thus accounts for only a low proportion of the polymorphism in this species.

In bacteria, the frequency of RecA-mediated homologous recombination depends on the level DNA identity, and small DNA fragments are often introduced into the cell via conjugation, transformation or transduction. Consequently, only a fraction of the genome might be targeted by recombination [47]. Several other factors might account for the low recombination frequency detected in the isolate of *R. etli *studied here. Among them, the ample degree of divergence among the studied *R. etli *strains, their distant geographical origins (USA, México, Costa Rica, Colombia, Brazil, and Spain) [24], and the small number of sampled strains. Recently, Bailly et al., reported a population genomics analysis of sympatric strains of *Sinorhizobium medicae *[27]. They found very low levels of polymorphism and recombination in the chromosome in comparison with the megaplasmids. Future studies using our methodology on *R. etli *isolates from single sites could be used to improve our understanding of how recombination impacts the diversification of this species.

## Conclusion

In summary, our results and the previous reports on *R. etli *support a model in which the species is composed of evolutionarily independent lineages that share a symbiotic phenotype but have low levels of recombination among the various lineages. However, although genetic barriers imposed by divergence or other barriers such as geographical distance might preclude homologous recombination among the strains, gene flow (e.g., by plasmids and chromosomal islands) is an ongoing process that shapes the genomic and pangenomic structures of *R. etli*.

## Methods

### Genomes used

Complete genome sequences were downloaded from GenBank as follows: for *R. etli CFN42*: chromosome [GenBank:NC_007761], and plasmids pCFN42a [GenBank:NC_007762], pCFN42b [GenBank:NC_007763], pCFN4c [GenBank:NC_007764], pCFN42d [GenBank:NC_004041], pCFN42e [GenBank:NC_007765], and pCFN42f [GenBank:NC_007766]; for *R. etli CIAT652*: chromosome [GenBank:NC010994], and plasmids pCIAT652a [GenBank:NC010998], pCIAT652b [GenBank:NC010996], and pCIAT652c [GenBank:NC010994]; and for *R. leguminosarum 3841*: chromosome [GenBank:NC_008380], and plasmids pRL7 [GenBank:NC_008382], pRL8 [GenBank:NC_008383], pRL9 [GenBank:NC_008379], pRL10 [GenBank:NC_008381], pRL11 [GenBank:NC_008384], and pRL12 [GenBank:NC_008378]. We also used reads and contigs from the draft genomes of *R. etli *strains 8C-3 [GenBank:NZ_ABRA00000000], BRASIL5 [GenBank:NZ_ABQZ00000000], CIAT894 [GenBank:NZ_ABRD0000000], GR56 [GenBank:NZ_AABRD0000000], IE4771 [GenBank:NZ_ABRD00000000], and KIM5 [GenBank:NZ_ABQY0000000].

### Determination of SNPs and pairwise nucleotide differences

Paired alignments between the draft genomes (contigs) and the ORFs from the genomes of CFN42 or CIAT652 were performed using the Dds2 program [48], which produces ungapped alignments of fragments having similarities greater than 80%. Each duplicated paired alignment (*i.e*., segments for which paralogous existed in the reference genome) was filtered using the reciprocal best hits option of the Fil program [48] under the following parameter set: coverage > 60% with respect to a reference gene and a percentage differential score cutoff < 10%. When two alignments had the same coverage, we selected the alignment with the higher score. Once the results were filtered, we created a gapped alignment using the Gap22 program [48] on segments for which the identity was greater than 85%. Both sequences were extracted using an *ad hoc *Perl script (homemade) formed for each paired alignment. To avoid frameshifts, we realigned each pair using cross-match [49] with the following parameters: discrep_lists masklevel, 0; tags gap_init, 3; gap_ext, 2; ins_gap_ext, 2; del_gap_ext, 2; minmatch, 14; maxmatch, 14; max_group_size, 20; minscore, 30; bandwidth, 14; and indexwordsize, 10. Finally, for each alignment, we determined the probability that a site was polymorphic using the Polybayes program [50], with the probability set at greater than 0.99 and a minimum Phred of Q45 [51,52].

### Assessment of methodological accuracy at low coverage

To determine if differences in coverage among the studied strains affected the reliability of the variability estimations, we took readings representing ~1× sequence coverages of seven *E. coli *genomes and complete-genome readings (about 10×) of the same genomes from GenBank (ftp://ftp.ncbi.nih.gov/genomes/) and assembled these readings using the Celera assembler [53]. The above-described analysis was applied to both the 1× and 10× coverage datasets, and the results were compared using the Mann-Whitney and Kolmogorov-Smirnov tests [33]. The utilized *E. coli *draft genomes were: 101-1 [GenBank:NZ_AAMK00000000], 53638 [GenBank:NZ_AAKB00000000], B171 [GenBank:NZ_AAJX0000000], E1100019 [GenBank:NZ_AAJW0000000], F11 [GenBank:NZ_AAJU0000000], HS [GenBank:NC_009800], and *O157_H7_ec4024 *[GenBank:NZ_ABJT0000000]. The complete genome sequence of *E. coli *K12 [GenBank:NC_000913] was used as the reference.

### Determination of triplets (homologous segments)

For the comparisons between all ORFs of the reference genomes (both CFN42 and CIAT652 were used throughout the work) and each incomplete genome (the contigs), we obtained the coordinates of all homologous segments (triplets) using the Mauve program [54]. Our analysis was standardized by aligning p42F (CFN42) against (*R. leguminosarum bv viciae 3841*) pRL12 using the following parameters: backbone-size = 100; max-backbone-gap = 50; weight = 90; island-size = 100. These plasmids were chosen because they contain shared syntenic blocks [36]. Sequence extraction, realignment of each conserved segment (backbone) and SNP determination were all performed as described above (see determination of SNPs section).

### Determination of quartets (orthologous segments)

To detect recombination events among DNA sequences, at least four sequences are required for the analysis [55]. Here, we first identified SNPs that distinguished each draft genome from the two reference genomes (CFN42 and CIAT652), and then determined the fragments that were shared between each draft genome and the ORFs from CFN42 and CIAT652, together with all replicons of *R. leguminosarum 3841 *(chosen because of its extensive synteny with CFN42) [36]. Sharing was determined using the Mauve program [54] (see determination of triplets section) and the shared fragments were realigned with the Muscle program (default parameters) [56]. To eliminate any large gaps within the alignments (rare in orthologous fragments), we used the Gblocks program under its default parameters [57].

### Detection of recombination

A variety of methods for detection of recombination have been reported in the literature [58], but no one strategy performs optimally under all evolutionary scenarios [59]. Therefore, a reasonable approach is to employ multiple methods and consider recombination events predicted by at least two methods as being the most reliable. Here, we used this strategy and considered recombination events that were detected by at least two of the following four programs [46]:

A) Geneconv [60]: Using this program, we ran 100,000 simulations for each quartet with the following parameters chosen: Dumptab; Dumpjseq; Dumpfrag; Annotate; WideCol; ShowBlast; Indel_blocs; ShowBcPwKaPvals; SortGfragsBySeq; Show_maxmeansims; ShowUnal; Gscale = 1; ListPair; ListBest; Bcsims; Allouter; Numsim = 100000/sp. This allowed us to detect possible genetic conversion events.

B) Pist [61]: With this program, we first identified the best-fit DNA substitution model for each shared fragment using the Akaike information criterion. We then used the best model to reconstruct the phylogeny using a maximum likelihood method (Phyml [62]) with 100 non-parametric bootstrap replicates. We next determined the invariant sites, alpha values, ts/tv ratios, base frequencies, and constant sites using the PAML program [63] and the GTR model. Finally, we ran Pist with the REV model and 10,000 permutations. Pist uses parsimony-informative sites to detect recombination events and is robust for highly divergent genes.

C) PhiPack [64]: We used the parameters of 10,000 permutations and a window size of 25 nt, and implemented the Pairwise Homoplasy Index, Maximum X2, and the Neighbor Similarity Score.

D) Hyphy program [65]: We used the routine GARD, which enables automated phylogenetic detection of recombination. We employed the GTR model and beta-gamma rate variation.

To determine if a recombinant gene was present in two different quartets or strains, we constructed a binary presence/absence matrix (1/0) for each gene that was found in two or more strains. These profiles were hierarchically clustered using the Cluster program [66].

### Phylogenetic analysis

Regions shared among all strains of *R. etli *and *R. leguminosarum bv viciae 3841 *were identified using the Mauve program [54], realigned by Muscle using the default parameters [56], and filtered for long gaps with Gblocks [57]. We then obtained the phylogeny of each region using a maximum likelihood approach employed by the Phyml program [62] (with 1,000 non-parametric bootstrap replicates) and the best nucleotide substitution model identified by the Akaike information criterion [67,68]. We used three methods to construct the phylogeny from the concatenated dataset, in order to determine the species tree. The first was the RAxML program (maximum likelihood) [69], in which we ran the GTR nucleotide substitution model and a GAMMA+P-Invar estimation of rate heterogeneity. This analysis yielded a Maximum Likelihood ML estimate of the alpha parameter and 1,000 distinct randomized Maximum Parsimony trees. The second program used was Phyml (maximum likelihood) [62], running 1,000 non-parametric replicates and the GTRG model. Finally, we employed the MrBayes program (Bayesian analysis) [70] running the Nucmodel 4by4 for DNA. The number of rate categories for the gamma distribution was set at four, with an allowance for a proportion of invariable sites. Because of the high computational burden, we performed two runs with four chains, for 500,000 generations in total. Trees were sampled every 500 generations, 25% of all samples were removed as reflecting burn-ins, and a consensus was obtained. Moreover, to assess differences in topology among the probable strain trees and individual gene trees, we used the Consel program [71], which calculated expected likelihood weighting and performed the Shimodaira-Hasegawa SH test [72]. Finally, a neighbor-net network was generated using the concatenated sequences and the Splits tree4 program [31].

### Nucleotide diversity and ts/tv ratios

For each shared fragment (quartet), we determined the nucleotide diversity and segregating sites using *R. leguminosarum 3841 *as an outgroup and employing the libsequence library [73]. The transition/transversion ts/tv ratios were determined for each quartet by using the PAML program [63] and applying the best model of nucleotide substitution obtained from each orthologous segment (see determination of quartet).

### Functional assignment

We used the COGs database [74] to undertake functional annotation across the four broad categories and sub categories to shared regions (all strains) as well as recombinant quartets. Quartets that had not been functionally assigned within the COG database were placed in the "Poorly Characterized'' category. For assignation to a category, we used the reciprocal best hits technique with an E-value < 1 × 10^-7^.

## Authors' contributions

JLA conceived, designed, and performed the experiments. JLA and VG analyzed the data and wrote the manuscript. RIS and PB were responsible for the genomic sequencing. PV, LEE and EM-R discussed the data. GD and VG contributed materials. VG edited the manuscript and is the Ph. D. thesis advisor of JLA. All authors read and approved the final manuscript.

## Supplementary Material

Additional file 1 **Strategy for Determining SNPs**. The additional file (in .pdf format) includes text and figures delineating our process for determining SNPs (parameters, paired comparisons and SNP differences). Also include the distribution of functional classes (COGs) of recombinant quartets of each draft genome and your comparison against distribution of CFN42. Click here for file

Additional file 2 **Table of genes presented in 240 shared regions**. The additional file (in .xls) includes the tables of genes and your features, as name, coordinates, gi, COGs and other. Click here for file
