# Supplementary material for: Genomic lineages of Rhizobium etli revealed by the extent of nucleotide polymorphisms and low recombination
Source: BMC Evol Biol. 2011 Oct 17;11:305. doi: 10.1186/1471-2148-11-305 (PMC3215678; doi:10.1186/1471-2148-11-305)

**Supplementary Material**

**Fig. 1** Assessment of parameters for determination of SNPs, using the Polybayes program. A) Bayesian probability of SNPs in raw readings, according to the Polybayes program output. Most SNPs (shown on a logarithmic scale) are polymorphic sites, with probabilities ≥0.975. Such patterns were seen in all incomplete genomes. B) Relationship between SNPs with probabilities of at least 0.975, and Phred quality values.


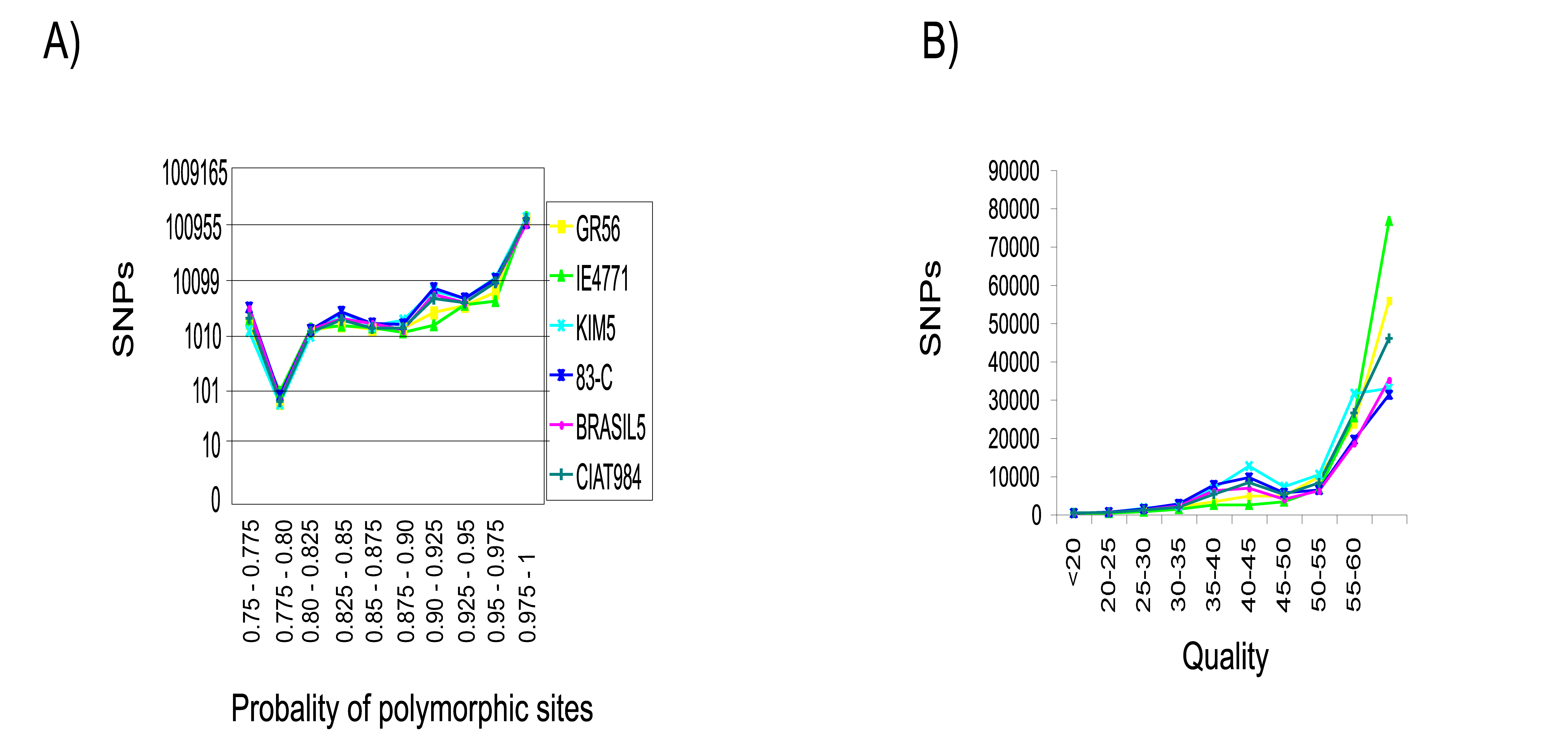


**Fig. 2** Comparisons of *Rhizobium leguminosarum viciae 3841* against the strains of *R. etli.* After using our methodology to identify SNPs, we determined the percentage of SNPs per gene fragment (Y axis). Boxes show the median values (middle line), and the first and third quartiles (lower and upper lanes), of the size distribution. The blue and red dots beyond the third quartile are outliers. Abscissa: all sampled strains of *R. etli*.


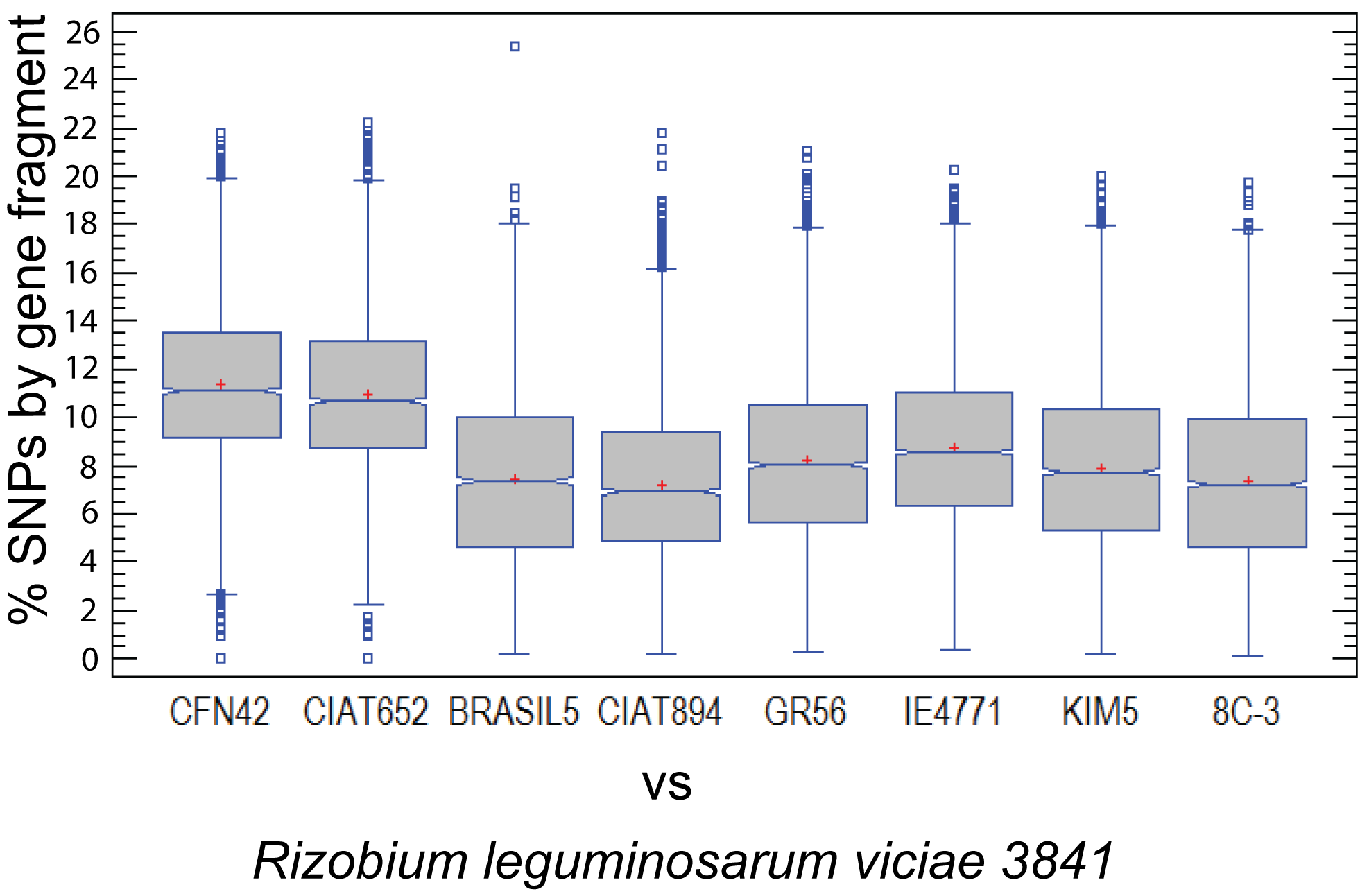


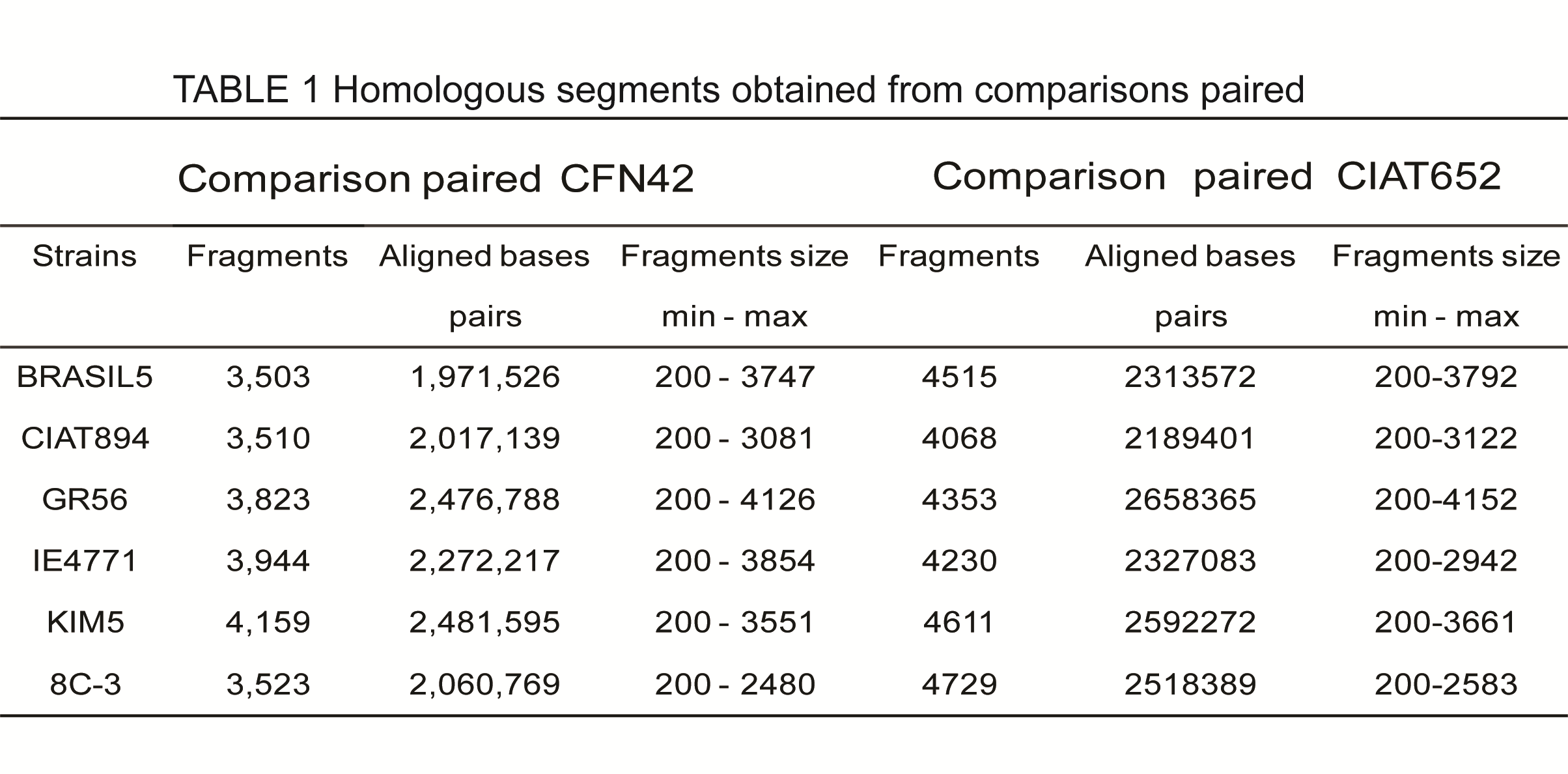


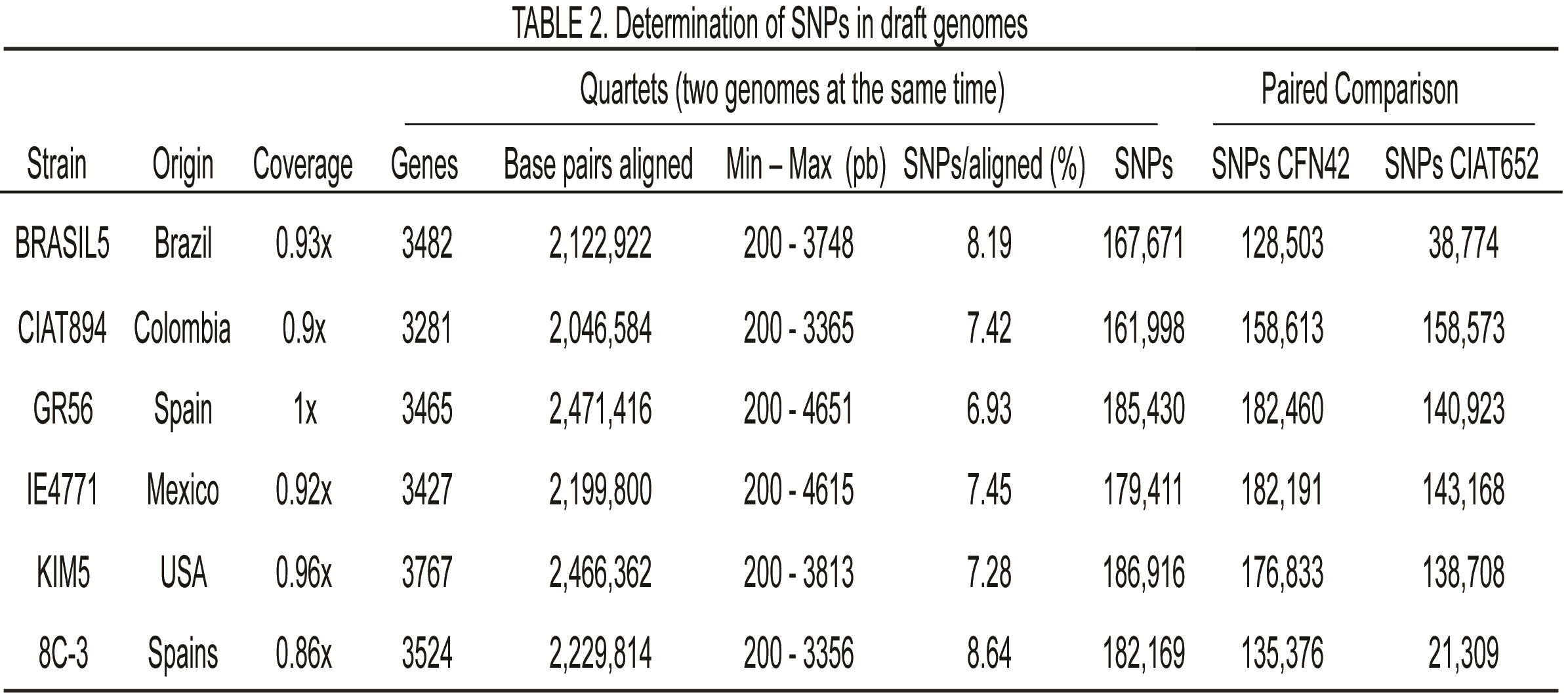


**Fig. 3** For each recombination event identified within of each quartet was assigned your functional annotation across the four broad categories and sub-categories found in the COGs database (Fig. 3). For assignation to a category, we used the reciprocal best hits technique with E-value < 1x10^-7^. Next, we quantified (relative frequency in the Y axis) each sub-category of COGs both for CFN42 and each draft genome. To observe if distribution of functional categories of recombinant quartets is different to categories presented in CFN42 genome, we compare each sub-category by chi-square and rank test used Predictive Analytics Software PASW Statistics 18 (SPSS Inc., Chicago, IL). The sub-categories with significant difference (p-value lower at 0.05) were indicated with asterisk.


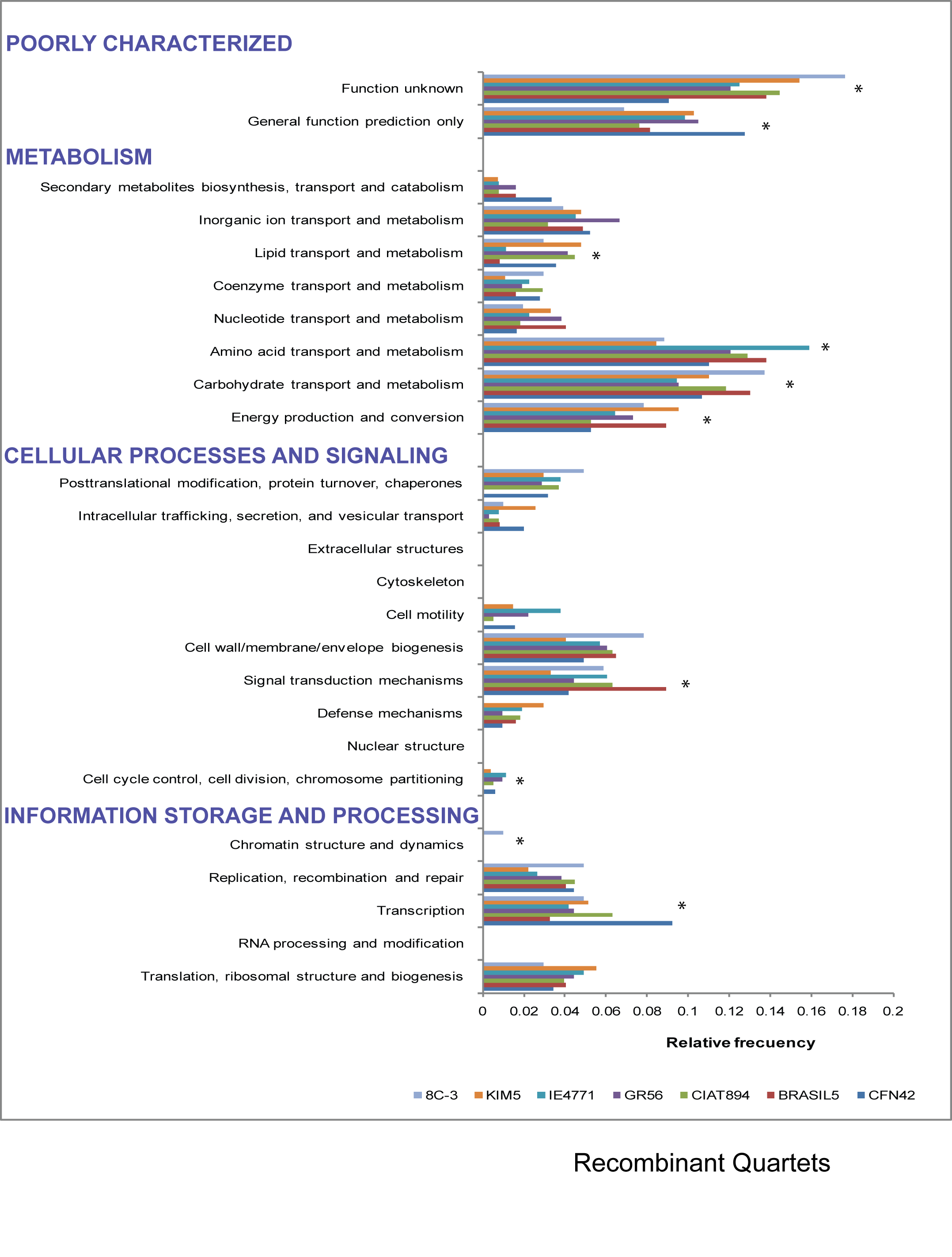

Supplement: Additional file 1 — Strategy for Determining SNPs. The additional file (in .pdf format) includes text and figures delineating our process for determining SNPs (parameters, paired comparisons and SNP differences). Also include the distribution of functional classes (COGs) of recombinant quartets of each draft genome and your comparison against distribution of CFN42. [file 1471-2148-11-305-S1.DOC]
